# An introduction to key performance indicators for medical physicists

**DOI:** 10.1002/acm2.13718

**Published:** 2022-07-13

**Authors:** Dominic J. DiCostanzo, Lalith K. Kumaraswamy, Jillian Shuman, Daniel C. Pavord, Yanle Hu, David W. Jordan, Christopher Waite‐Jones, Annie Hsu

**Affiliations:** ^1^ Department of Radiation Oncology The Ohio State University Columbus Ohio USA; ^2^ Department of Radiation Oncology Novant Health Cancer Institute Charlotte North Carolina USA; ^3^ Department of Radiology Ascension Via Christi Saint Francis Wichita Kansas USA; ^4^ Department of Radiation Oncology Allegheny Health Network Pittsburgh Pennsylvania USA; ^5^ Department of Radiation Oncology Mayo Clinic Arizona Phoenix Arizona USA; ^6^ Departments of Radiation Safety and Radiology, University Hospitals Cleveland Medical Center, Department of Radiology Case Western Reserve University Cleveland Ohio USA; ^7^ Centennial Medical Physics Fort Collins Colorado USA; ^8^ Department of Medical Physics, Edmond Odette Cancer Centre Sunnybrook Health Sciences Centre Toronto Ontario Canada

**Keywords:** clinical physics, key performance inidicators, patient safety, quality control

## Abstract

Qualified medical physicists (QMPs) are in a unique position to influence the creation and application of key performance indicators (KPIs) across diverse practices in health care. Developing KPIs requires the involvement of stakeholders in the area of interest. Fundamentally, KPIs should provide actionable information for the stakeholders using or viewing them. During development, it is important to strongly consider the underlying data collection for the KPI, making it automatic whenever possible. Once the KPI has been validated, it is important to setup a review cycle and be prepared to adjust the underlying data or action levels if the KPI is not performing as intended. Examples of specific KPIs for QMPs of common scopes of practice are provided to act as models to aid in implementation. KPIs are a useful tool for QMPs, regardless of the scope of practice or practice environment, to enhance the safety and quality of care being delivered.

## 1 INTRODUCTION

According to Medical Physics Practice Guideline (MPPG) 10, “Qualified Medical Physicists (QMPs) are members of multidisciplinary health care teams. As medical specialists, QMPs are dedicated to ensuring the safety of patients, medical staff, and the general public and improving quality of care to ensure accurate diagnosis and treatment of disease.^1^” The primary goal of health care is the safe and effective treatment of patients with the highest standards and efficiencies. With rapid technological advances and treatment options, health‐care providers are constantly striving to improve the quality and safe delivery of care. Key performance indicators (KPIs) are a critical component to effectively monitor, evaluate, and continuously improve the performance of health services.^2^, ^3^ KPIs are quantifiable measures of performance for a specific objective.^4^ A well‐designed KPI can be utilized to monitor performance in health‐care services and can provide actionable insight into the activities from which they are derived. As a result, choosing the correct measures is of the utmost importance and requires significant forethought. The goal of well‐designed KPIs is to provide meaningful information that can be used to monitor and improve the quality and safety of the services provided.^5^, ^6^

KPIs are often considered in the domain of business administration, but recent publications have shown their applicability to medicine.^7^ In addition, health care is adopting more business‐style approaches to administering departments, such as lean operations, six‐sigma quality, and tracking revenue‐generating versus nonrevenue‐generating activities. As such, KPIs have been adopted throughout hospitals and health‐care systems, but there has been limited implementation by specific departments, such as radiation oncology, radiology, and nuclear medicine. As KPIs can be designed to evaluate a departmental process or they can be designed to measure the performance of specific teams within the department, such as QMPs,^8^ an opportunity exists to provide guidance to QMPs concerning the creation and application of KPIs. For example, the number of patients delayed from starting their treatments in a radiation oncology department is a measure of a generic KPI, whereas the number of patients delayed from starting their treatments due to delay in patient‐specific quality assurance (QA) is a measure of a KPI specific to medical physics. Providing this type of guidance is in‐line with the efforts of the American Association of Physicists in Medicine (AAPM) Medical Physics 3.0 (MP3.0) initiative.

MP3.0 is an initiative to aid QMPs in reevaluating the traditional practice patterns associated with medical physics.^9^ MP3.0 highlights the expertise of QMPs in four major areas: clinical, science, education, and leadership. KPIs are a tool that when implemented support the QMP's progress toward goals in each of these domains. The scope of KPIs, as applied to these domains, may seem unmanageable when looked at from a large perspective, but MPPG 10 highlights many specific activities that overlap with the domains of expertise highlighted by MP3.0.^1^, ^9^ By breaking down the domains into smaller and smaller portions, as done with Appendix A of MPPG 10, it is possible to develop KPIs that would be applicable to all QMPs regardless of the scope of practice (therapy, diagnostic, nuclear medicine, magnetic resonance imaging, or medical health physics), practice environment, or individual responsibilities.

This document is structured as an introduction to the subject of KPIs for QMPs from definition to implementation. We share some examples of KPIs that may be applicable to the reader or at least guide the development of KPIs specific to the reader's interests and environment. The previous literature includes additional examples that the reader is encouraged to review that span the breadth of practice environment and scope of practice.^4^, ^5^, ^6^, ^10^, ^11^ This document is not meant to be an exhaustive text on KPIs, and the reader is encouraged to perform further study on KPIs.

## 2 FUNDAMENTALS OF KEY PERFORMANCE INDICATORS

### 2.1 Definition

KPIs are quantitative metrics used to track progress toward an end goal. When well crafted, KPIs provide actionable insight toward the desired outcome.^12^ This requires KPIs to change over time and do so at frequent enough intervals that interventions can occur if needed. KPIs can track any number of variables related to the goal, but it is imperative that there is a target value for each indicator; otherwise, the KPI is reporting data without context or insight.

The intended stakeholders of the indicators will play a role in development, as different audiences will find certain information more applicable. For instance, consider the case of development of KPIs for a hospital administrator versus a chief physicist. In the case of the hospital administrator in radiation oncology, it may be important to use patient treatments per day due to the revenue generated by each fraction delivered. The administrator may use fractions delivered to consolidate daily budgets and track progress toward the year‐end revenue. However, for a chief physicist, this information may be less relevant. Instead, it may be more illuminating to have a KPI for the percentage of QA tasks completed in a given period allowing the chief physicist to track progress toward the goal of 100%. In radiology, an administrator may review the percent uptime of equipment, whereas a physicist is more interested in machine performance. By combining these two interests, KPIs could be generated to monitor routine QC for trends that can predict machine downtime and allow more time to conveniently schedule preventive maintenance.

### 2.2 Utility/applicability

Just as it is important to design the appropriate KPI for a given audience, KPIs developed in a vacuum by QMPs will limit the use and relevance to a broader audience. Although KPIs can be used to track physics‐specific measures, it is important to consult with all leaders in the department when creating more publicly visible KPIs. Utilizing the input from others will provide two specific benefits: understanding the needs of others who will be using the KPIs and promoting acceptance of the KPIs when developed using a team approach.

Utilizing the expertise and understanding the needs of those in leadership positions within the department will provide insight and identify the metrics that show the value of a QMP. Depending on the practice environment or subspecialty, the KPIs will vary, but the goal is to understand the current blind spots that exist. Once the blind spots are identified, the creation of KPIs can occur with the merging of clinical and administrative knowledge. This integration will provide more accurate and actionable KPIs than those developed independently by a QMP. Additionally, a team approach promotes increased interest and investment from others leading to a higher likelihood of adoption and use.^13^

For example, in radiology, administrators, physicists, and biomedical engineers may generate a KPI that monitors equipment uptime. During annual equipment evaluations, follow‐up on corrective action may result in downtime. By tracking how often follow‐up is required, administrators, physicists, and biomedical engineers can work together to decrease downtime. Additional preventative maintenance may be required by biomedical engineers, closer monitoring of QC by physics may be necessary, or changes to staff workflow may be necessary if issues are caused by mistreatment of equipment (e.g., current cleaning solution may be harming equipment). Additionally, this KPI could help administrators target machines that need to be replaced soon and avoid the downtime. All three stakeholders are directly impacted by this KPI and have a vested interest in its outcome.

### 2.3 Creation

In all practice environments, QMPs play a central role in ensuring safety. It is evident that this role would lend to the creation of KPIs that target quality and safety. To do so effectively, key stakeholders and users of the KPIs will need to be identified. Once identified, the stakeholders can discuss the needs and prioritization of indicators. Each stakeholder can develop their own list of KPIs for their specific area and then aggregate them for a leadership team to evaluate and choose.^14^ However, once the topics for KPIs are decided upon, it is important that all participants recognize KPI development as a group effort that reflects the needs of each stakeholder.

Once the areas of focus have been identified, the definition and refinement of the KPIs can begin. This process should be iterative and requires thought and planning. The goal of the indicator is similar to continuous quality improvement initiatives. The indicators should allow for opportunities to intervene and adjust as needed. Here is an example of a therapy physics KPI that has little value: Percentage of Plans this Month Receiving Physics Check Prior to Treatment. This KPI provides little opportunity for intervention. By aggregating and requiring an update only once per month, there is little information that can be gleaned from the metric as to the operations on a regular basis. Second, as the report by AAPM Task Group 275^15^ states

*The review of radiotherapy treatment plans and charts by a qualified medical physicist is a key component to ensuring safe, high‐quality care. … It is called for in numerous society‐level recommendations [e.g., American Association of Physicists in Medicine (AAPM) TG‐40, ACR‐ASTRO Guidelines]*.

As a result, one would fully expect that the KPI would show 100% success rate without taking into consideration the potential delays in care that a patient may have if the check is not performed in a timely manner. It provides no information as to the workflow of the department. One may rightly ask, “How long did the QMP allow the chart to idle prior to beginning the check?” or “How close in time to the scheduled treatment did the physician approve the plan?” Finally, this KPI would provide no information regarding the quality of the work performed by the QMP; rather, it provides only time‐based information.

In addition to these specific flaws in the example, understanding how the underlying data is gathered to generate a KPI is important. Data collection for KPIs should be automatic, when possible, to avoid unnecessary use of additional resources. In the aforementioned example, using database queries to gather date and time stamps of the first treatment compared to the completion of the physics check would be minimally invasive and have high fidelity, while still suffering from the previous weaknesses. Requesting that the QMP log completion of a physics check in addition to the completion of the required documentation would add additional work and should be avoided whenever possible.

Additional strain on the system to generate KPIs should be avoided for a variety of reasons, but two are of consequence and worth mentioning. Fidelity of data is of paramount importance when being applied to KPIs. If manual data entry is required to generate the KPI, then there is a chance of incorrect data entry or data omission. With the goal of the KPI providing actionable information, any deviation could lead to unnecessary interventions that may lead to unintended consequences. Second, observer bias can occur if data are collected manually leading to possible serious side effects in the use and application of the KPIs. If the underlying data is unreliable, then the decisions made that rely upon the KPI will be faulty. This could be as severe as process changes that create inefficiencies or decrease safety, or as minor as creating mistrust in the implemented KPIs.

In radiology, radiation dose index monitoring software can be used to assist in generating KPIs with limited additional burden to the physicist. When technologists manually record CT or fluoroscopy dose indices, the rate of error can be high resulting in additional work for the physicist to track down the actual machine reported dose. Dose index monitoring software has the benefit of receiving structured dose reports directly from the machine, which results in higher fidelity of data and the capability of acquiring additional data that would be too burdensome for the technologist to record, including technique factors. This results in a more robust and reliable KPI.

In radiation oncology, mitigating data fidelity and observer bias in addition to automating data collection can be managed by extracting data from oncology information systems (OIS). OISs are built on relational databases that provide detailed date and time information among other variables. KPIs created using the data contained can leverage automatic data collection and lack of observer bias. Data fidelity can still be a challenge but can be overcome with robustly designed processes and well‐maintained databases.

Along with the data collection methodology, the frequency of data collection should be decided at the onset. For systems or processes that may change rapidly, an increased frequency of data refresh may be needed. However, for long running projects or long‐term goals, it may not be intuitive as to the rate needed to ensure that the KPIs are providing useful information. It is incumbent upon the group designing and developing the KPIs to consider this during conceptualization.

The last step in the development of a KPI is to set a target value for each indicator. This step requires an understanding of the system, process, or goal that may not be available to all stakeholders. It is the responsibility of those who have the domain knowledge to appropriately set target values that align to the system or process. If chosen wisely, the target value should be a representative of value that will show success or progress, which may be challenging to the current process. The target can motivate those utilizing the KPI toward a common goal with clear purpose or to communicate the goal. The targets should be specific, measurable, action oriented, realistic, and time based.^16^

### 2.4 Implementation

After the initial development of a KPI, it is important to test the KPI using real data for which the QMP already has a clear understanding of the progress or outcome linked to it. This pilot phase provides a validation of the KPI and its implementation phase to ensure that there are no gross miscalculations taking place. The stakeholder group can review the KPI and its target value to ensure the appropriateness of the metric as well as the target value. Once the validation of the specific metric has been performed, it is important to implement a periodic review cycle.

The stakeholder group must determine at what frequency to review the metric, which includes the target value and underlying data. Early in implementation, this review may need to be more frequent to ensure the validity of all aspects of the KPI. As time progresses, it is appropriate to review with reduced frequency, but it is important to review at some interval to ensure applicability to current goals and validity of the target value. It is necessary to review KPIs after process changes to ensure that the indicators are either unaffected or affected (positively or negatively), requiring a target value adjustment.

KPIs are meant to be monitored continuously as they provide actionable insight and should align closely with operational and strategic goals. Transparency of KPIs is important if the team‐performing actions, which feed into the KPIs, are to be motivated by projected goals. Without visibility to the measures being tracked, it is unlikely any changes will occur. However, it is possible that the stakeholders will implement small changes to evaluate their effect on a specific KPI. In this case, it is still important that the KPIs remain visible to provide transparency and increase awareness.

During the monitoring phase, it is beneficial to create and use action levels, akin to ALARA Levels in radiation safety or tolerance and action levels for those familiar with the report by AAPM Task Group 142.^17^ These levels identify additional values beyond the target value that are relevant to the monitoring process and stakeholder group. For instance, an action level may be set if a KPI value exceeds the target value by a set amount and signal the need to reevaluate the target level. An action level may also be set to flag a KPI that is below the target value by a certain amount or for a period of time signaling the need for a specific intervention.

## 3 SUMMARY

Developing KPIs requires the involvement of stakeholders in the area of interest. Fundamentally, KPIs should provide actionable information for the stakeholders using or viewing the KPIs. During development, it is important to think through the underlying data collection for the KPI, making it automatic whenever possible. Once the KPI has been validated, it is important to set up a review cycle and be prepared to adjust the underlying data or action levels if the KPI is not performing as intended. QMPs ensure “the safety of patients, medical staff, and the general public and improving quality of care to ensure accurate diagnosis and treatment of disease.^1^” As a result, QMPs are in a unique position to influence the creation and application of KPIs across diverse practices in health care. KPIs are a useful tool for QMPs, regardless of the scope of practice or practice environment, to enhance the safety and quality of care being delivered.

### 3.1 Key performance indicator examples

1. Diagnostic Medical Physics—Computed tomography (CT) dose index monitoring
2. Therapeutic Medical Physics—Patient‐specific QA gamma pass rates
3. Diagnostic Medical Physics—Imaging equipment performance evaluation (EPE) findings rate
4. Therapeutic Medical Physics—Final chart check timing compliance

### 3.2 Computed tomography dose monitoring

**KPI title**: CT dose index monitoring.

**KPI description**: Monitor computed tomography dose index–volume (CTDI_vol_) to look for protocol changes and potential out‐of‐range studies.

**KPI rationale**: CT dose represents a tradeoff between image quality and patient safety. A CTDI_vol_ that is low may indicate that a study that does not have sufficient image quality to make a diagnosis. Alternatively, a patient may be exposed to unnecessarily high radiation beyond what is needed to achieve a diagnostic‐quality exam. It is important to monitor CTDI_vol_ for trends that can indicate that a protocol has been changed, resulting in a potential change in image quality and patient dose. The Joint Commission requires that organizations review and analyze incidents where a radiation dose index, such as CTDI_vol_ exceeded expected dose index ranges identified in imaging protocols.

**KPI target**: CTDI_vol_ for a given scan should be less than the dose notification levels recommended in AAPM Dose Check Guidelines version 1.0^18^ and should exceed a minimum threshold level set by the site. All scans with a CTDI_vol_ outside of this range are considered out of range.

**KPI calculation**: CTDI_vol_ is calculated by the scanner and included in the structured dose report. This scanner‐reported value is verified annually by a QMP.

**Data source**: Radiation dose structured reports sent directly from CT scanner to database.

**Data collection frequency**: Daily.

**Minimum data set**: Scanner‐reported CTDI_vol_, DLP, reference phantom used, study date, patient age, and accession number.

**External comparison**: ACR dose index registry (DIR) achievable dose levels and diagnostic reference levels.

**KPI monitoring**: Reports shall be generated monthly, and any out‐of‐range scans will be investigated by a QMP. On a quarterly basis, the CT Protocol Committee shall review the reports for potential trends and changes.

**KPI reporting frequency**: Monthly reports with out‐of‐range scan summary shall be sent to site managers. Quarterly CT protocol review shall be performed by the CT Protocol Committee.

An example of a KPI for the diagnostic use of CT is a radiation dose index in CT. Effective CT dose index monitoring and evaluation allow us to continuously improve the performance of health services. The CT dose index as a KPI may also reveal issues in protocols, software, and hardware. CT dose index information is readily available and can be pulled directly from the dose report. Therefore, it is a good candidate for the KPI. Based on the clinical practice of individual institutions, CT dose index information can be updated at a preselected frequency (daily, weekly, etc.). This information would be reviewed periodically to ensure consistent performance. It is also recommended to benchmark institution data against ACR aggregate data to identify opportunities for improvement.

**FIGURE 1:**
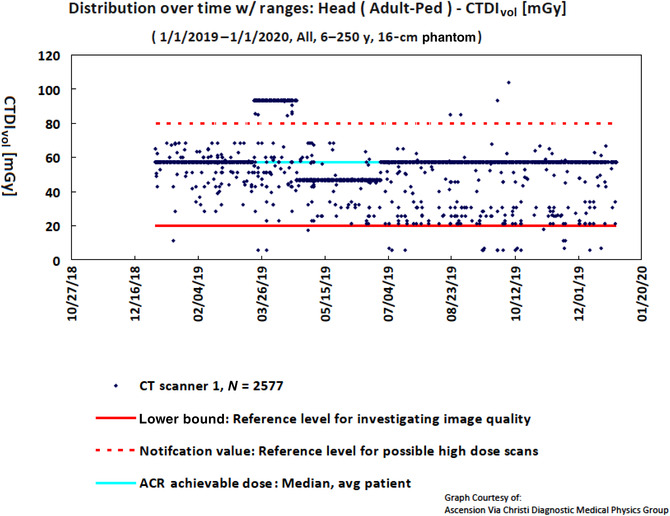
The computed tomography dose index–volume (CTDI_vol_) of patients scanned using head protocols from 01/01/2019 to 01/01/2020

As an example, Figure 1 provides CTDI_vol_ of patients scanned using head protocols from 01/01/2019 to 01/01/2020. A large shift in the CTDI_vol_ was noticed in April 2019. After investigation, it was found that it was due to a modified protocol. After the protocol was fixed, CTDI_vol_ values returned to the range among the reference levels.

**FIGURE 2:**
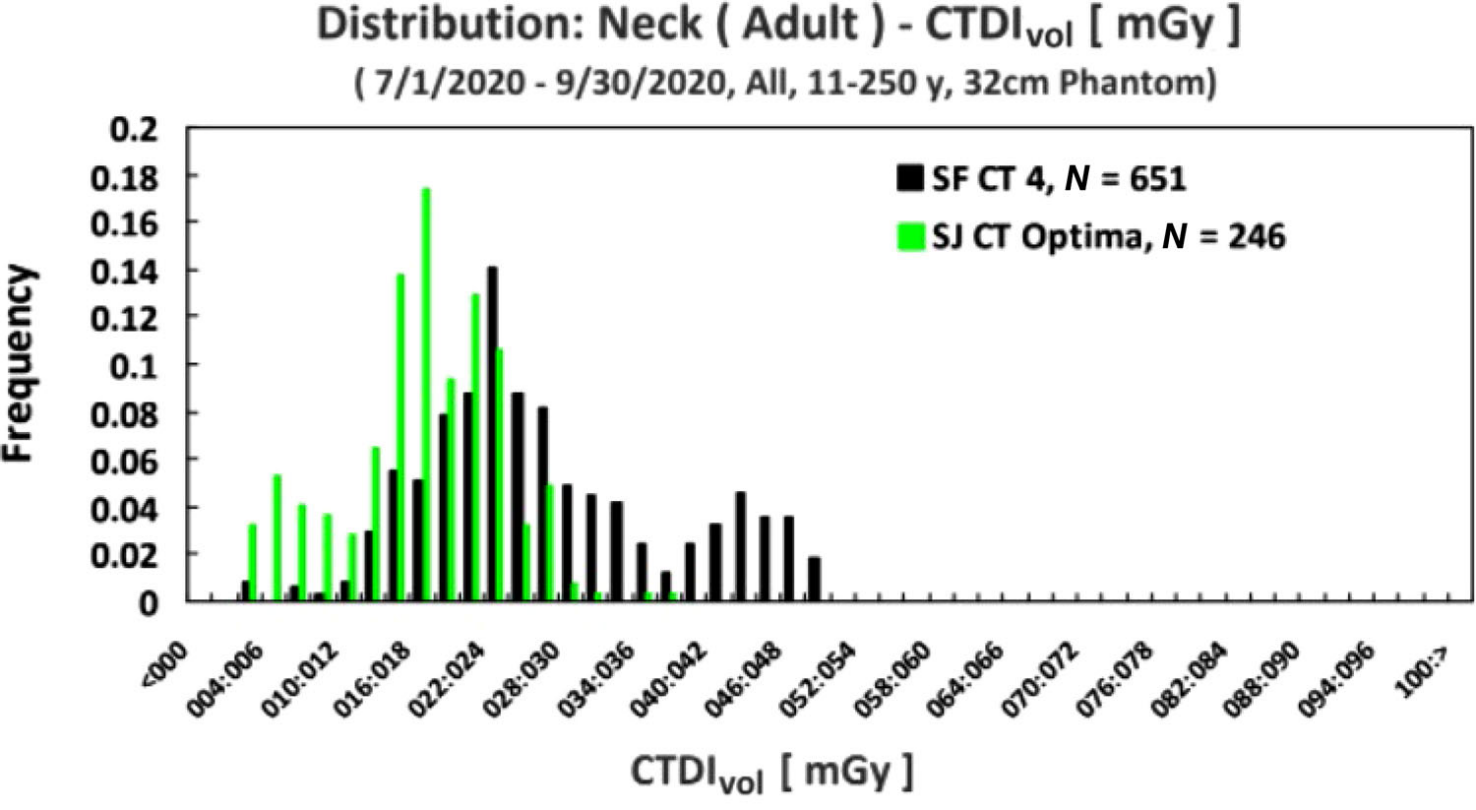
Histogram of computed tomography dose index–volume (CTDI_vol_) for neck protocols for two separate CT scanners from 07/01/2020 to 09/30/2020

As a second example, during monthly review physics noted that the average CTDI_vol_ for neck protocols was substantially different on one scanner versus another that was the same exact make and model. Figure 2 shows that the SF scanner has a bimodal distribution. The distribution surrounding the 46‐mGy mark was the average dose before the protocol was changed to be the same as the SJ scanner. After a brief investigation, it was noted that the noise index on the SF machine was much lower than the SJ machine.

**FIGURE 3:**
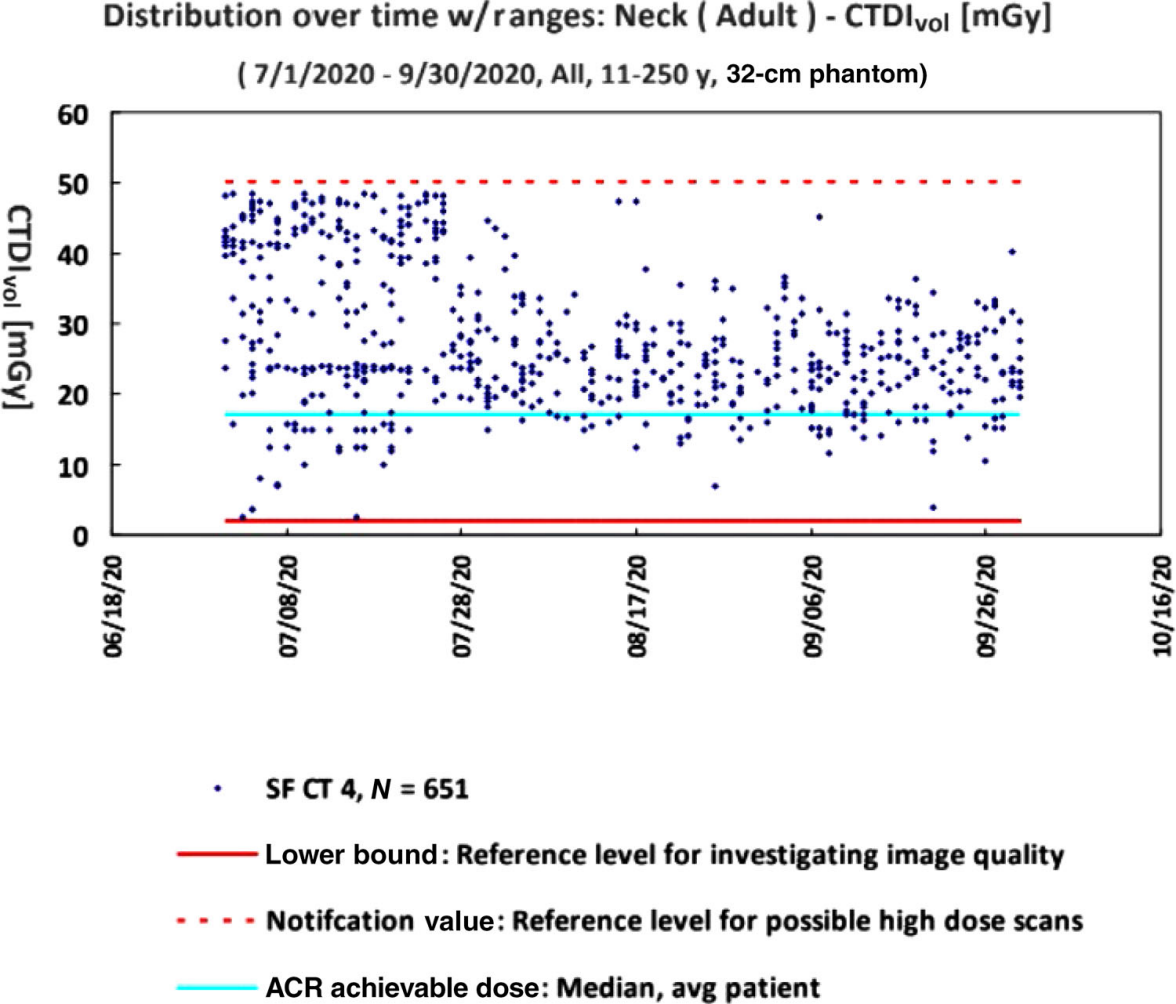
The computed tomography dose index–volume (CTDI_vol_) of patients scanned with neck protocols from 07/01/2020 to 09/30/2020

In discussions with the radiologist on the CT Protocol Committee, it was determined that the protocols and image quality should be the same for SF as SJ. Figure 3 shows how the dose changed after the protocol was modified on 7/26/2020.

### 3.3 Patient‐specific quality assurance gamma pass rates

**KPI title**: Monitoring patient‐specific QA gamma pass rates.

**KPI description**: Monitor the trend in gamma pass rates to detect TPS system changes and to detect linac performance issues, especially multi‐leaf collimator (MLC) performance.

**KPI rationale**: Patient‐specific QA is performed on intensity‐modulated treatment fields to ensure that the calculated plan dose can be delivered accurately without any errors. According to AAPM Task Group report on Tolerances and Methodologies for IMRT QA (TG 218), gamma pass rate of ≥95% indicates as good deliverable treatment plan.^19^ Monitoring the trend in gamma pass rates over time could indicate a change in the TPS or deterioration of linac components, especially the multi‐leaf collimation system.

**KPI target**: Patient‐specific QA should yield a gamma pass rate ≥95%, with 3%, 2 mm, and a 10% threshold.

**KPI calculation**: Gamma pass rate is calculated according to the methodology illustrated by Low et al.^20^ Currently the software tools are available to automatically calculate the gamma pass rates.

**Data source**: Patient‐specific QA document attached to each patient's treatment record.

**Data collection frequency**: Obtained for all IMRT/VMAT treatments.

**Tracer conditions**: Gamma pass rate and DTA.

**Minimum data set**: Patient identifier, gamma pass rate, date of QA, and measuring device.

**KPI monitoring**: Reports can be generated monthly and evaluated by a QMP for any trends that show pass rates falling below the tolerance limit or approaching the limit.

**KPI reporting frequency**: Monthly.

An example of KPI in radiation therapy is the gamma pass rate obtained from performing patient‐specific QA on radiation treatment plans. Gamma pass rates indicated the deliverability of the treatment plan to achieve the clinically intended goals. Patient‐specific QA is performed on intensity‐modulated treatment fields to ensure that the calculated plan dose can be delivered accurately without any errors. Several investigators have provided metrics and action levels to assess the validity of the treatment fields against the predicted dose.^21^, ^22^, ^23^, ^24^, ^25^ AAPM TG 218 recommends the tolerance limit for gamma pass rate should be ≥95%, with 3%/2 mm and a 10% dose threshold.^19^ Patient‐specific QA allows not only to assess the deliverability of the radiation treatments, but also to monitor the MLC performance. Patient‐specific QA pass rates for individual treatment plans can be collected and monitored for a period as shown in the figure given later. Continuous monitoring of the patient‐specific QA pass rate can be a KPI that allows us to detect any MLC performance issues as well as changes in the treatment planning system.

**FIGURE 4:**
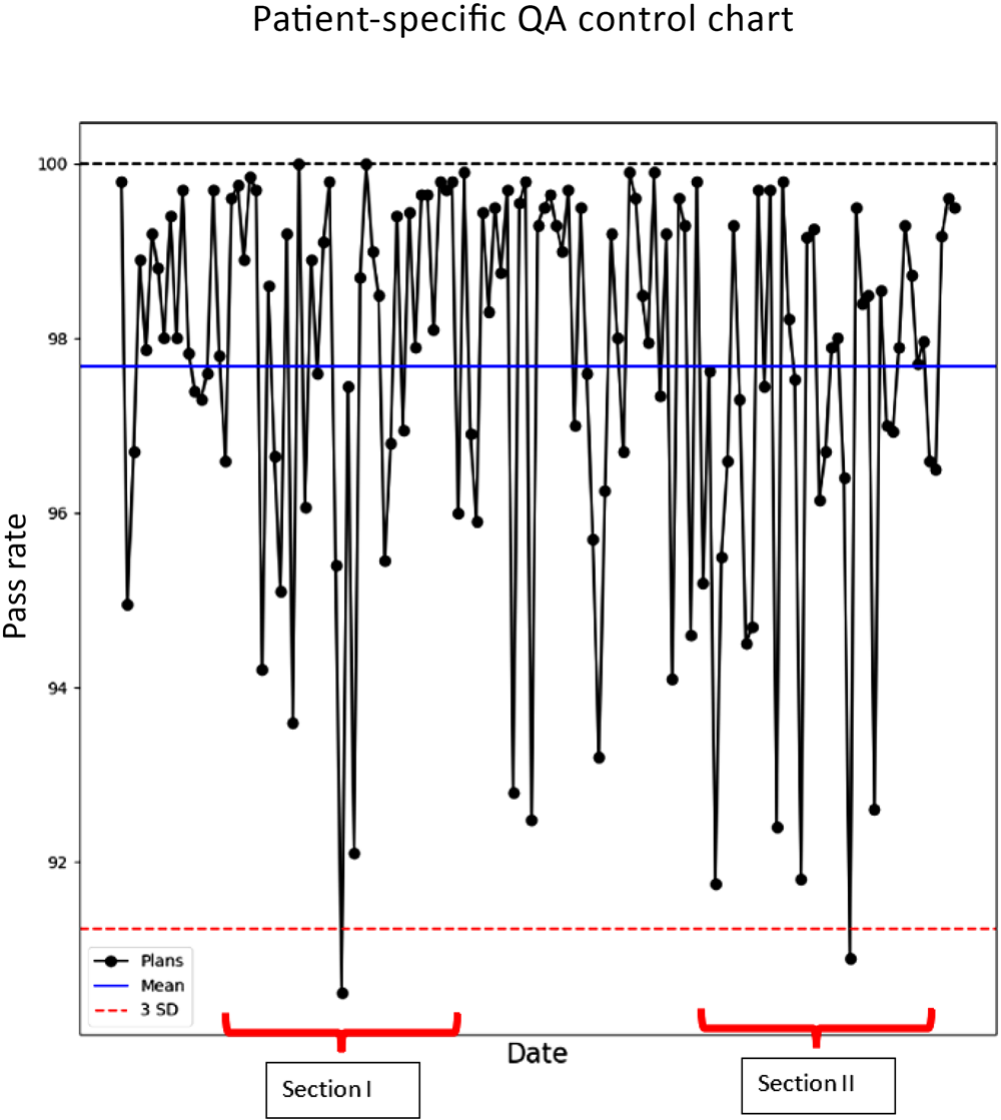
Patient‐specific quality assurance gamma pass rates as a function of time. Sections I and II highlight plans that neared and exceeded three standard deviations from the mean pass rate prompting investigations.

Figure 4 provides the gamma pass rates over several months for a Varian TrueBeam Linac (Varian Medical Systems, Palo Alto, CA) equipped with Millennium 120 MLC leaves. In Sections Ι and ΙΙ, treatment plans fell below or came close to the tolerance limit. Further investigation of Section Ι revealed that the maximum MLC leaf speed parameter in the TPS was increased during a TPS software upgrade. This resulted in MLC leaf position errors in several highly modulated treatment fields and hence the patient‐specific pass rates fell below the tolerance limit. After adjusting the maximum MLC leaf speed parameter to an acceptable value in the TPS, the failure rate decreased dramatically. Investigation of Section ΙΙ revealed that two of the outer MLC leaf motors (Leaf A55 and B53) were failing. Treatment fields involving these outer MLC leaves had lower pass rates compared to the fields not employing these leaves for dynamic treatment delivery. After replacing the leaf motors, the gamma pass rates for subsequent QA increased.

The use of measurement‐based pretreatment QA has been the source of much debate. As new guidance emerges on the specifics of what is the most effective QA methodology, the type of monitoring described here could be adapted to any new testing regimen.

### 3.4 Imaging equipment performance evaluation findings rate

**KPI title**: Imaging EPE findings rate.

**KPI description**: Percentage of annual EPE with recommendations for follow‐up corrective actions.

**KPI rationale**: The overall rate of findings in an installed base of equipment justifies the rationale for performing annual evaluations. Deviations from the baseline rate by modality, facility, or physicist may indicate practice improvement opportunities. Findings often indicate needed repairs or calibrations that would not have been detected or completed without the physics EPE.

**KPI target**: A baseline findings rate is established for a physicist group and an installed base of equipment undergoing routine evaluations. The KPI target is to maintain a findings rate consistent with the baseline. Changes to physics personnel or the equipment inventory may require a new baseline.

**KPI calculation**: $\frac{N_{f}\left(t\right)}{N_{EPE}\left(t\right)}$

*N_EPE_* is the total number of EPEs conducted during the period *t*, and *N_f_* is the number of EPEs with actionable findings conducted during the time *t*.

**Data source**: Reports of findings for annual physics surveys.

**Data collection frequency**: Monthly to annually.

**Minimum data set**: Date and results of EPE (findings or no action required).

**External comparison**: Baseline value for initial monitoring period and rates of other groups/practices.

**KPI monitoring**: QMP or medical physics assistant shall enter actionable findings in a shared spreadsheet after each EPE is completed.

**KPI reporting frequency**: Monthly review for potential trends by a QMP. Annual review for overall equipment concerns.

An example of a KPI for diagnostic and nuclear medicine physics practices would be the fraction of annual EPEs resulting in recommendations for follow‐up corrective action. These may take the form of actions needed by the department or facility staff or repairs or adjustments to the equipment by qualified service personnel. Each type of equipment undergoes a set of specific tests appropriate to the equipment and its clinical use following established procedures. In addition, the QMP typically reviews the results of the ongoing QC (e.g., daily, weekly, or monthly) and makes recommendations regarding the result data and any corrections to the procedures. Findings on an annual EPE report with recommendations for corrective action indicate nonoptimal operating conditions that may have continued undiscovered if the EPE had not been conducted.

**FIGURE 5:**
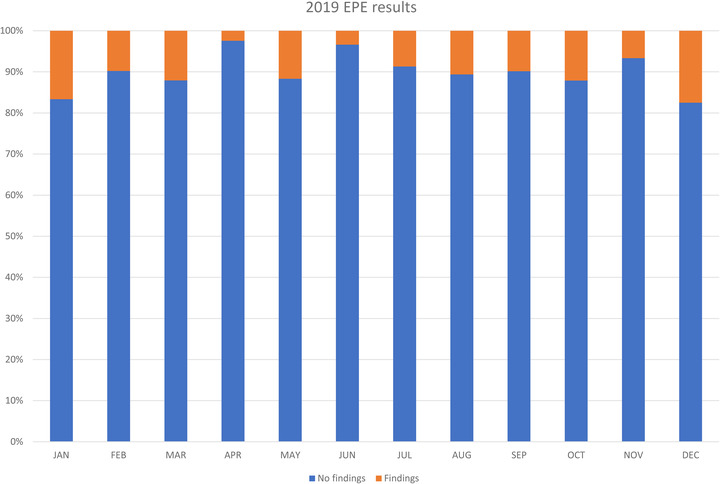
Percentage of annual equipment performance evaluations with need for follow‐up corrective actions for calendar year 2019

As an example, Figure 5 shows monthly EPE results for the calendar year 2019 for a medical physics service group. Each month shows the total number of EPEs performed and the number resulting in actionable findings. The overall rate of EPEs with findings for the year was 10%.

The rate of EPEs with findings requiring corrective action can be determined for each modality or equipment type, different individual QMPs, and individual facilities supported by the group, comparing each to the overall rate and the baseline rates.

### 3.5 Final chart check timing compliance

**KPI title**: Final chart check timing compliance.

**KPI description**: Monitor the adherence to the timetable of completing final treatment chart reviews.

**KPI rationale**: Final chart checks are recommended by accrediting organizations^26^ and the AAPM (TG315 not yet published). The final chart check to ensure accurate delivery of the prescription should be performed within 1 week of the end of treatment. This is to ensure the attending physician is alerted to any deviations with the intended treatment plan.

**KPI target**: The target for this KPI would be that all patient charts are checked by a QMP within 7 days from the end of treatment.

**KPI calculation**: To calculate compliance, the length of time, in days, from the completion of patient treatment to physics closeout is calculated for each patient course:

$$\Delta T=T_{closed}-T_{Tx}$$

where for a given reporting period Δ*T* is the time from final treatment to physics chart closeout, *T_Tx_* is the date of final treatment, and *T_closed_* is the date of the physics closeout.

**Data source**: Automatically pulled from the electronic health record data.

**Data collection frequency**: The data is refreshed ad‐hoc or at specified intervals with 1 month chosen as a specific refresh cycle.

**Minimum data set**: Patient's medical record number, date of closeout, and date of final treatment.

**KPI monitoring**: Reports can be generated at a desired interval (e.g., monthly) and emailed automatically to the QMP group or chief physicist.

**KPI reporting frequency**: Monthly.

An example of a KPI for therapeutic medical physics practices would be to check compliance with the length of time between patients’ final treatment that receive a final chart check. According to accrediting bodies^26^ and AAPM TG315 (not yet published), all patients should receive a final chart check at end of treatment to validate that the treatment was delivered as intended within 1 week from treatment completion. Any deviations from the prescription should be escalated to the radiation oncologist. To ensure compliance with the accrediting standards and best practices, it is possible to use the electronic health record (or record and verify system) to automatically track compliance.

**FIGURE 6:**
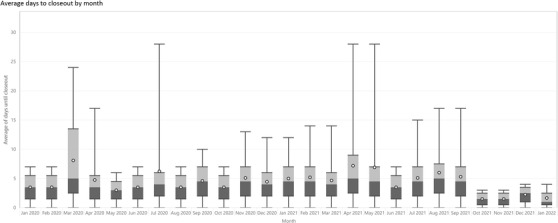
Box and whisker plots for the average days until closeout from final treatment in the date range 01/01/2020 to 01/31/2022. The decline in time to complete closeouts in October 2021 was related to the implementation of new processes.

As an example, Figure 6 shows the data for 2 years from a QMP practice. The box and whisker plots for each month display the distribution in the length of time, including the maximum, from treatment conclusion to closeout. Beginning in October 2021, new processes were introduced, whereby QMPs performed closeouts daily rather than a weekly, greatly reducing the variability and maximum length of time.

The length of time between treatment conclusion and closeout can be a useful tool in ensuring compliance with accreditation standards and best practices. The automated data collection and reporting add minimal overhead to tracking compliance.

## AUTHOR CONTRIBUTION

Dominic J. DiCostanzo initially conceived the idea, performed literature review, collected data for therapy examples, drafted text and figures, and revised the document.

Lalith K. Kumaraswamy participated in the initial scoping and outline of the document, performed literature review, collected data for therapy examples, drafted text and figures, and revised the document.

Jillian Shuman participated in early discussions regarding topic, provided diagnostic physics input, collected data for diagnostic examples, drafted text and figures, and revised the document.

Daniel C. Pavord participated in early discussions regarding topic, assisted with literature review, drafted text and figures for therapy examples, and revised the document.

Yanle Hu participated in early discussions regarding topic, drafted text and figures for diagnostic examples, and revised the document.

David W. Jordan developed framework, collected data, drafted text and figures for a diagnostic example, and revised the document.

Christopher Waite‐Jones participated in early discussions on the topic, helped to draft text and figures for diagnostic examples, and provided review and revision of the document.

Annie Hsu participated in early discussions on the topic as well as provided critical review and revision of the document.
